# Subaxial cervical foraminal chondromas: case-based discussion on surgical management

**DOI:** 10.1007/s10143-024-03065-w

**Published:** 2024-11-04

**Authors:** Alberto Vandenbulcke, Andrea Sanjurjo, Anne-Laure Rougemont, Sana Boudabbous, Rodolfo Maduri

**Affiliations:** 1 Department of Clinical Neurosciences, Unit of Neurosurgery, Lausanne University Hospital, Lausanne, Switzerland; 2 Division of Clinical Pathology, Viollier Laboratory, Geneva, Switzerland; 3 Diagnostic Department, Division of Clinical Pathology, Geneva University Hospital, Geneva, Switzerland; 4 Diagnostic Department, Service of Radiology, Geneva University Hospital, Geneva, Switzerland; 5 Department of Orthopedics and Traumatology, Hôpital Riviera Chablais, Rennaz, Switzerland

**Keywords:** Spinal neoplasm, Chondroma, Spinal cord, Vertebral body, Cervical vertebrae, Spinal nerve root, Radiculopathy

## Abstract

Cervical foraminal chondromas are benign lesions that may require surgical resection when symptomatic due to radicular and/or spinal cord compression. The aim of surgery is to achieve gross tumor removal while preserving neurological function and spine stability. The authors describe a case of subaxial foraminal chondroma with a systematic review of the literature on patients with cervical chondromas. In the reported case, the authors used a retrojugular approach to remove a C6-C7 right chondroma without the need for spinal stabilization. Literature review identified a total of 11 patients who underwent surgery for subaxial foraminal chondroma. The mean age at diagnosis is 33.6 years (range: 10–73). Most patients report neurological symptoms at the time of diagnosis. The most frequently involved vertebral level is C4-C5 (54.6%, 6/11). Preoperative foraminal enlargement is present in 63.6% (7/11) of patients. Surgical resection is performed via an anterior approach in 18.2% (2/11) of patients, with vertebral body resection and concomitant cervical instrumentation. The anterolateral approach is selected in 27.2% (3/11) of patients, and the posterior approach in 54.6% (6/11) of patients, with only one patient requiring both anterior and posterior instrumentation. The choice of surgical access for subaxial foraminal chondroma can be challenging due to the anatomical location of the tumor in relation to the cervical nerve roots and spinal cord. Accurate approach selection is key to achieving complete tumor removal while preserving cervical spine stability.

## Introduction

Foraminal cervical tumors account for 6–18% of subaxial cervical spine neoplasms [1, 28]. Peripheral nerve sheath tumors, namely schwannomas and neurofibromas, are the most frequent subtypes of subaxial cervical spine tumors with prevalent foraminal locations, accounting for 71–97% of all foraminal lesions [17, 34], while chondromas are encountered in 4% of the patients [21, 22, 28]. Despite their benign histology, subaxial cervical chondromas may exhibit locally aggressive behavior with rapid growth and consequent nerve root compression, mass effect on the spinal cord and surrounding neck structures. Symptomatic foraminal chondromas require complete resection, but the appropriate surgical strategy remains debated due to the sparse literature [1, 3–5, 10–12, 18, 23, 27, 28].

In the present paper, the authors report a clinical case of foraminal chondroma and conduct a systematic review of the literature on subaxial foraminal chondromas. The aim is to describe the clinical and diagnostic features of subaxial chondromas and treatment outcomes to support decision-making for the management of these rare spine tumors.

## Materials and methods

A comprehensive systematic review of the literature was is conducted on the PubMed database up to June 2024 to identify foraminal chondroma of the subaxial cervical spine. PRISMA (Preferred Reporting Items for Systematic Review and Meta-Analysis Protocol) guidelines are followed. Articles are selected using a Boolean search with the keywords “chondroma” OR “periosteal chondroma” AND “cervical spine.” Two reviewers (A.V. and R.M.) independently select the pertinent articles. Articles in English, French, and Italian are considered for further selection. Only studies reporting histologically confirmed chondromas (periosteal or central) of the cervical spine (from C3 to C7) and foraminal invasion are selected. Patients of all ages are included.

The following data are obtained from the review of the publications that met the inclusion criteria:

- Preoperative demographic variables (age, sex), presence of neck pain, radiculopathy, myelopathy, symptoms of neck compression (dysphagia, hoarseness, Horner syndrome);
- Surgical approach defined as follows:
  - Anterior approach: Cloward approach to the cervical spine with or without partial or complete corpectomy [13].
  - Anterolateral approach: access to the neural foramen medially to the internal jugular vein [5, 18].
  - Posterior approach: standard midline approach with hemilaminectomy, bilateral laminectomy with or without partial or complete facetectomy [12, 22].
- Postoperative data: extent of tumor resection (GTR: gross total resection; STR: subtotal tumor resection, biopsy); extent of bone resection (laminectomy, corpectomy, facetectomy). The authors also report spine fusion and adjuvant treatment (radio- or chemotherapy). Tumor recurrence was reported, as well as the clinical outcome at follow-up defined with the McNab scale (Excellent, good, fair, and poor) [20].

Patients characteristics are describe with the following variables: frequency and percentage for categorical data, mean and range for continuous data.

**Fig. 1:**
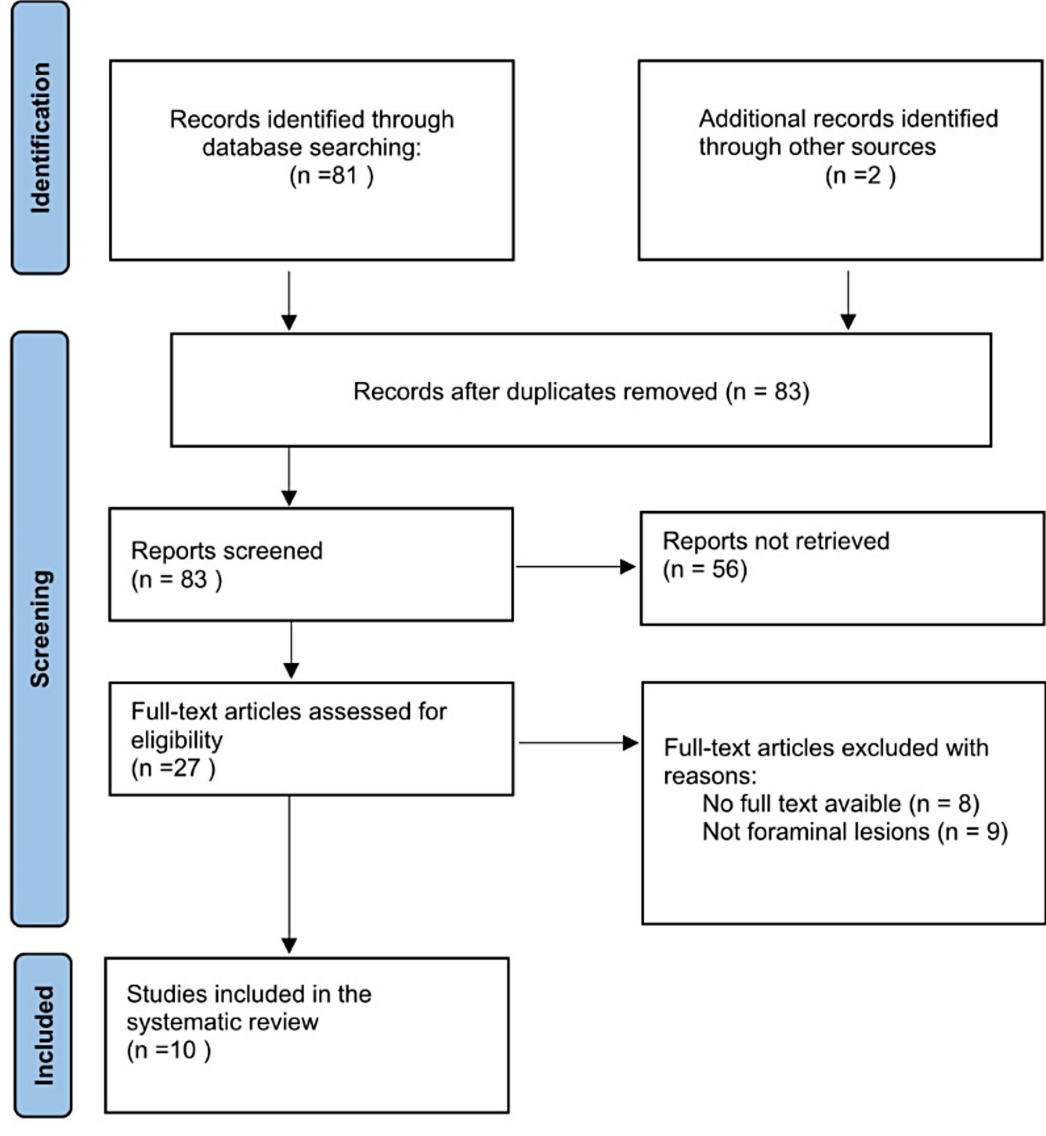
The article selection process for the systematic review of the literature

The primary PubMed search identifie s81 studies. The article selection process is shown in Fig. 1. The initial screening based on title and abstract analysis leads to the inclusion of 28 studies. Two more studies are identified through the bibliography of the analyzed articles. A further detailed review of the available studies identifie s10 articles matching the inclusion criteria [2, 9, 14, 19, 25, 29, 31–33, 36]. All studies are evaluated as being of good quality based on the tool proposed by Murad et al. [26]. All of these are case reports. The authors also include their case report in the literature review.

## Results

### Case example

The authors report the case of a 49-year-old female who presents a recent worsening neck pain and right C7 radiculopathy, with a visual analogue scale (VAS) for arm pain at 10/10 despite using tramadol and pregabalin. The Neck Disability Index (NDI) at presentation is 25/50 (50%), indicating severe impairment. The neurological examination shows right triceps palsy with muscular strength at M4/5 on the Medical Research Council (MRC) scale.

**Fig. 2:**
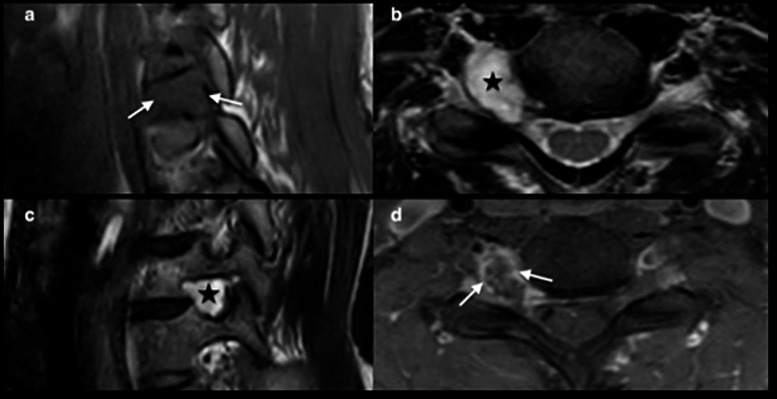
Pre-operative cervical MRI. Panel **a**: Sagittal T1-weighted images; Panel **b**: Axial T2-weighted images; Panel **c**: Coronal oblique T2-weighted image; Panel **d**: Axial T1 Fat sat weighted image after contrast injection

**Fig. 3:**
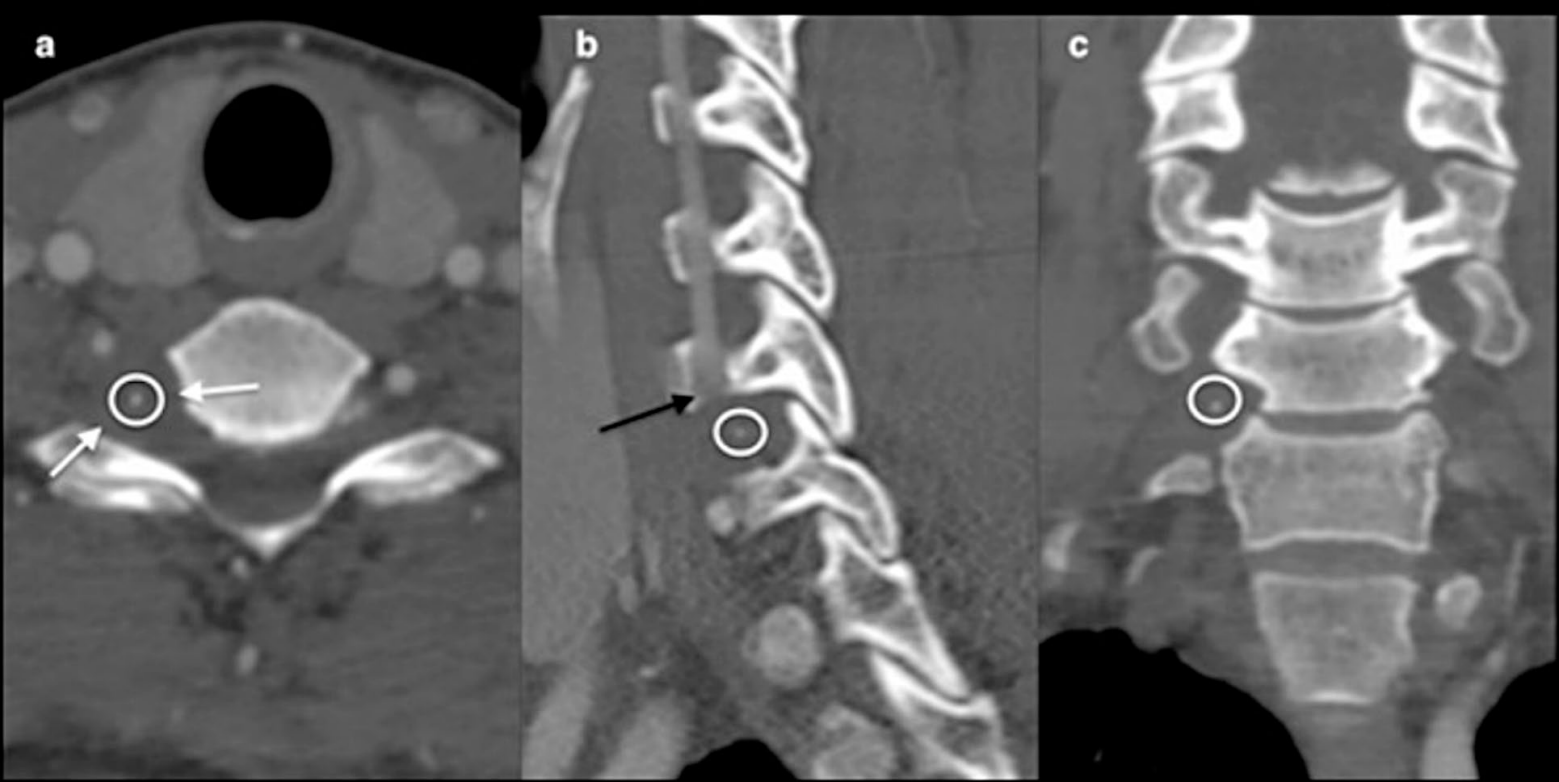
Cervical CT shows a right C6-C7 foraminal lesion with intralesional calcifications (white circle). Panel **a**: axial images. Panel **b**: sagittal images, the black arrow shows a right C6-C7 foraminal mass in close relationship with the vertebral artery without arterial obstruction. Panel **c**: coronal images

Cervical MRI shows a cystic mass at the right C6-C7 foramen with peripheral ring enhancement (Fig. 2). A CT-angiogram reveals a close relationship between the tumor and the V1 segment of the vertebral artery (VA) without significant arterial stenosis, but with enlargement of the right C6-C7 foramen and intralesional calcifications (Fig. 3). Due to severe disability and the neurological deficit, the patient underwent surgical resection. Due to the foraminal localization with predominant ventral extension and foraminal widening, the authors opt for a retrojugular approach. This approach provides direct access to the lesion with minimal bone resection and offers good control of the ventrally displaced VA.

The description of the retrojugular approach is the following: through a skin incision extending along the anterior border of the sternocleidomastoid muscle (SCM), a subplatysmal flap is raised anteriorly until the midline and posteriorly up to the trapezius. The SCM is mobilized to expose the carotid sheath and, superiorly, the spinal accessory nerve. The internal jugular vein (IJV) is skeletonized and mobilized along with the vagus nerve. The transverse cervical artery, scalene muscles, phrenic nerve, and trunks of the brachial plexus are then identified. The V1 segment of the vertebral artery (VA) is also identified but not mobilized or transposed.

The C6-C7 foramen is identified under fluoroscopy, and the anterior part of the C7 transverse process is removed under robotic exoscopic visualization (Modus V, Synaptive Medical) using an ultrasound bone scalpel (Sonopet, Stryker) to access the neural foramen. Due to an error of localization, the C6 transverse process was removed instead of C7, causing brisk VA bleeding. The arterial bleeding is controlled using patties and coagulation at 20 Malis units, 3 watts for less than a second to progressively close the arterial breach. After verification with fluoroscopy, the C7 transverse process was identified and removed using the ultrasound scalpel.

After visualization of the foraminal tumor, careful drilling of the anterior margin of the C6 and C7 vertebral bodies permits the enlargement of the neural foramen. Piecemeal (intralesional) resection is accomplished under robotic exoscope magnification using standard microsurgical techniques. The tumor appears covered by periosteum, with the right C7 nerve root identified and preserved. The tumor is resected through the neural foramen with no intradural portion identified. At the end of the procedure, the resection is considered with positive margins [8]. No fusion is required.

In the immediate postoperative period, the patient presents regression of the right C7 radicular pain and improvement of the triceps palsy (M4+/M5 MRC). As a complication, the patient developed right Horner’s palsy. Postoperative CT angiogram shows no right VA dissection with complete canalization of the vessel.

**Fig. 4:**
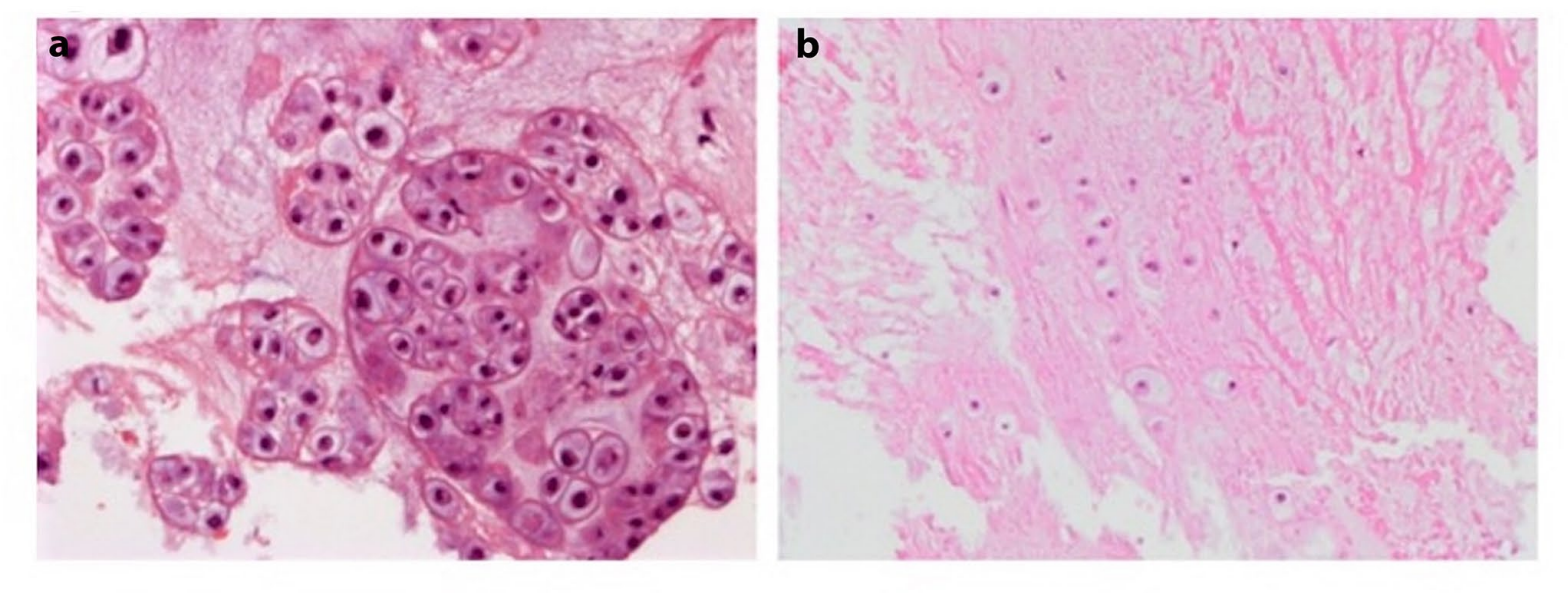
Histology (x200; hematoxylin-eosin stain) reveals a purely well-differentiated cartilaginous tumor, composed of small lobules of bland chondrocytes (Panel **a**) alternating with paucicellular areas showing chondrocytes arranged in isogenic columns (Panel **b**)

Histology (Fig. 4) shows a well-differentiated cartilaginous lesion with small lobules of chondrocytes embedded in a chondroid, partially myxoid matrix. Cellularity is slightly variable, but the chondrocytes display a small nucleus with no significant atypia; no mitotic activity and no necrosis are seen. The immunohistochemical study shows cytoplasmic reactivity to S100 protein and nuclear SOX9 reactivity, consistent with the cartilaginous nature of the tumor. There is no reactivity to epithelial markers (AE1/AE3, EMA) and brachyury. The definitive diagnosis is in favor of juxtacortical chondroma or enchondroma.

**Fig. 5:**
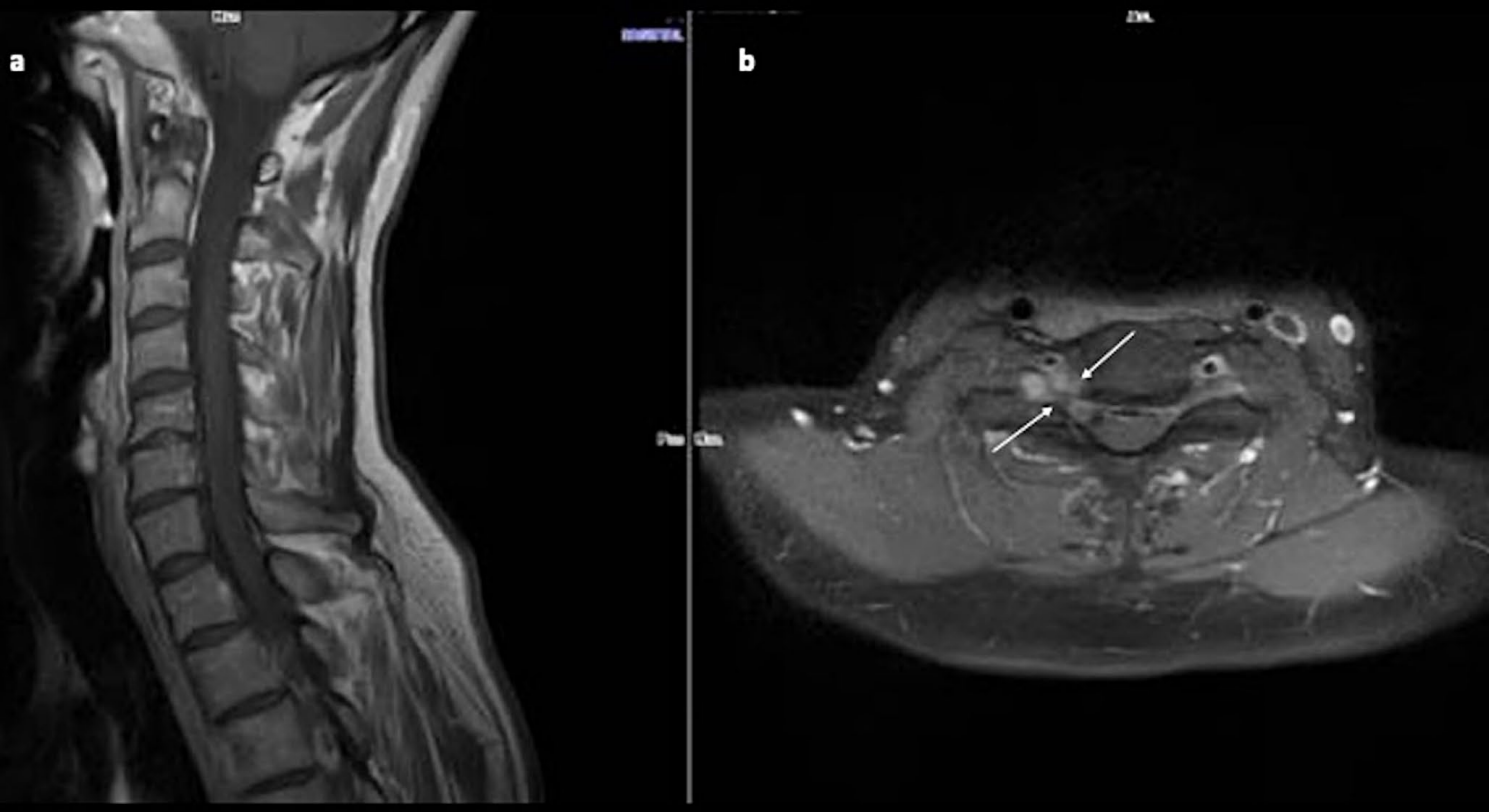
Follow-up cervical MRI with contrast-enhanced T1 sequences (Panel **a** sagittal, Panel **b** axial views) showing absence of tumor recurrence at the C6-C7 right foramen (white arrows)

At 36 months of follow-up, the patient is pain-free with complete recovery of right triceps palsy and partial regression of the right Horner’s syndrome. The NDI score at 3 years is 3/50 (6%). Follow-up MRI (Fig. 5) shows the right C6-C7 foramen with no tumor recurrence.

### Systematic review of literature

The literature review identifies a total of 10 patients who underwent surgery for subaxial foraminal chondroma [2, 9, 14, 19, 25, 29, 31–33, 36], with the addition of the 11th case reported by the authors. The patients’ characteristics are outlined in Table 1. The mean age at diagnosis is 33.6 years (range: 10–73), and gender distribution shows a female prevalence (F = 7:4). Most patients report neurological symptoms at diagnosis. The clinical manifestation is radiculopathy in 33% (4/11) and myelopathy in 16% (2/11), while a combination of radiculopathy and myelopathy is present in 33% (4/11). 18% (2/11) of patients present with symptoms related to tumor mass effects on the surrounding neck structures (dysphagia, hoarseness, and cervical mechanical pain).

The most frequently involved vertebral level is C4-C5 (54.6%, 6/11), followed by C5-C6 (18.2%, 2/11), C6-C7 (18.2%, 2/11), and C7-T1 (9%, 1/11). Preoperative foraminal enlargement is present in 63.6% (7/11) of patients. Surgical resection is performed via the anterior approach in 18.2% (2/11) of patients with vertebral body resection and concomitant cervical instrumentation. The anterolateral approach is selected in 27.2% (3/11) of patients and the posterior approach in 54.6% (6/11) of patients, with only one patient requiring both anterior and posterior instrumentation. Gross total resection (GTR) is achieved in 81.1% (9/11), in one patient (9%) subtotal resection (STR) is decsribed and in one patient the extent of resection is not evaluable. No patient received adjuvant treatment (radio- or chemotherapy). The mean follow-up duration is 18 months (range: 3–42 months). All patients treated with GTR are considered as cured at follow-up with no tumor recurrence, while the only patient with STR presenting tumor recurrence 12 months postoperatively thus undergoing second surgery. For the patients with available follow-up data (10/11, 90.9%), McNab’s score is evaluated as excellent in 60% (6/10), good in 30% (3/10), and poor in one patient (10%).

**Table 1:** Clinical presentation and management of cervical foraminal chondromas

Author (Year)	Slowik T (1968)[33]	Willis BK (1986)[36]	Lozes G (1987)[19]	Palaoglu S (1988)[29]	Baber WW (1988)[2]	Morard (1993)[25]	Shurland AT (1999)[32]	Fahim DK (2009)[9]	Jeong D (2005)[14]	Sarmiento JM (2019)[31]	Maduri (2024)
Age (years)	10	23	73	30	50	39	11	6	24	55	49
Sex	F	F	F	M	M	M	F	M	F	F	F
Clinical manifestation	Myelopathy	Radiculopathy	Radiculopathy + Myelopathy	Radiculopathy + Myelopathy	Radiculopathy + Myelopathy	Myelopathy	Neck mass	Dysphagia	Radiculopathy	Radiculopathy + Myelopathy	Radiculopathy
Level	C6-C7	C4-C5	C4-C5	C5-C6	C4-C5	C7-T1	C4- C5	C5-C6	C4-C5	C4-C5	C6-C7
Foraminal enlargement (Yes/No)	Yes	NA	Yes	NA	Yes	Yes	No	Yes	Yes	No	Yes
Surgical approach	Posterior	Anterior	Antero-lateral	Antero-lateral	Posterior	Posterior	Posterior	Anterior	Posterior	Posterior	Antero-lateral
Extent of bone resection	Laminectomy	Corpectomy	Uncal resection	NA	laminectomy + facet resection	Laminectomy + pediccle resection	Hemilaminectomy	Corpectomy	Hemilaminaectomy + partial fecetectomy	Laminectomy	N/A
Instrumentation (Yes/No)	Yes (Post)	Yes (Ant)	Yes (Ant)	NA	No	No	No	Yes (Ant + Post)	No	Yes (Ant + Post)	No
Extent of resection	GTR	GTR	GTR	STR	GTR	GTR	NA	GTR	GTR	GTR	GTR
Histological diagnosis	Chondroma	Enchondroma	Bening Chondroma	Chondroma	Bening Periosteal Chondroma	Chondroma	Enchondroma	Chondroma	Enchondroma	Juxtacortical Chondroma	Periosteal Chondroma
Adjuvant treatment	No	No	No	No	No	No	No	No	No	No	No
Follow-up (months)	42	12	36	12	24	3	NTA	3	36	12	36
Tumor recurrence (Yes/No)	No	No	No	Yes (re-op)	No	No	NTA	No	No	No	No
McNab scale	Excellent	Excellent	Excellent	Failure	Good	Excellent	NA	Good	Excellent	Good	Excellent

Legend: Ant: anterior, Post: posterior F: female, M: male, GTR: gross total resection, NA: Not available, STR: subtotal resection

## Discussion

Foraminal chondromas of the subaxial spine are prevalent in women in their third decade (mean age at diagnosis: 33.6 years). Their clinical manifestations can include mechanical neck pain, cervical radiculopathy and/or myelopathy. The presentation of foraminal chondromas at diagnosis is non-specific and similar to other lesions such as disc herniations [17]. Despite their rarity, chondromas should be considered in the differential diagnosis of cervical foraminal lesions. While MRI can easily differentiate disc herniations and osteophytic spurs from foraminal tumors, the differential diagnosis remains challenging. Chondromas have non-specific MRI features, presenting as lobulated T1 and T2 isointense or hypointense lesions with peripheral contrast enhancement [30]. CT scans of subaxial spine chondromas may show intralesional calcifications, as seen in the present case. Foraminal chondromas are benign lesions that can be differentiated into two histological subtypes according to their growth pattern in the vertebral body: (1) central chondromas or enchondromas (ECs), and (2) periosteal (juxtacortical) chondromas or ecchondromas (PCs) [21, 24]. ECs are located in the spongious cavities of the vertebral body and may lead to pathological fractures as they grow. PCs arise from the periosteum of the vertebral body, resulting in cortical bone thickening. Despite their benign histology, foraminal chondromas may exhibit rapid growth, causing symptomatic neural and vascular compression. The present review shows that gross total resection (GTR) for subaxial chondromas is curative, while subtotal resection (STR) has a high risk of tumor recurrence, necessitating repeat surgery [7, 25]. The overall clinical outcomes for patients operated on foraminal chondromas are favorable in 90% of cases, underscoring the importance of accurate diagnosis and appropriate surgical management.

Surgery for foraminal chondromas can be challenging, and the appropriate surgical approach must be defined based on several variables: lesion extension, presence of bone resorption, cervical instability, and the relationship of tumors with the spinal cord and surrounding neck structures. Different surgical approaches are described in the literature for the removal of foraminal chondromas: (a) anterior, (b) posterior (laminectomy), or (c) antero-lateral/retrojugular approaches.

- **Anterior surgical approach**: Through a standard Cloward procedure, this approach requires extensive bone removal for adequate tumor exposure, posing a risk of spine instability. The anterior cervical approach for foraminal chondromas is often associated with various degrees of vertebrectomy, necessitating systematic spinal instrumentation. Therefore, the anterior approach should be reserved for patients with osteolytic chondromas involving the vertebral body and/or extending ventrally to the spinal cord [13]. An anterior approach and corpectomy were described in two cases in our review (Table 1). Fahim et al. and Willis et al. performed multi-level corpectomies for large foraminal lesions with significant vertebral body involvement [9, 36].
- **Posterior approach (laminectomy)**: This familiar procedure for most surgeons requires the resection of at least two adjacent facet joints to achieve adequate tumor exposure, with a significant risk of spine instability. All patients operated on foraminal chondromas through unilateral or bilateral laminectomy received at least two-level facetectomies, necessitating spinal instrumentation [1, 12, 15, 16, 23, 27]. These findings align with previous reports showing that cervical instability almost systematically occurs in patients after unilateral resection of at least two facet joints [16, 27]. Furthermore, the posterior approach carries a potential risk of VA and nerve root injury due to the lack of direct visualization during resection [1, 27]. This approach should be reserved for lesions with extensive intracanal involvement, lateral spinal cord displacement, and posterior element invasion, with no or minimal vertebral body erosion, that are inaccessible through an anterior approach[2, 14, 25, 31–33]. A variable degree of bony resection may be performed, as demonstrated in the following two cases. Jeon et al. performed a hemilaminectomy and partial facetectomy without the need for instrumentation for a C4-5 foraminal enchondroma with foraminal enlargement, unilateral posterior element involvement, and lateral spinal cord displacement [14]. Morard et al. reported a case of a C6-T1 lesion with bilateral foraminal invasion and anterolateral displacement of the spinal cord. Due to the posterior and foraminal extension, the case was successfully treated using a multi-level laminectomy and facetectomy[25].
- **Retrojugular approach**: This approach allows direct access to the neural foramen through the antero-lateral route to the cervical spine. First described by Verbiest [35] and popularized by Lot and George [18], it is considered a safe anatomical corridor to cervical foraminal lesions. During the retrojugular approach for cervical chondromas, limited bone removal after foraminal tumor resection allows access to the intradural tumor component [18, 35] while preserving spine stability [5]. The VA and cervical nerve roots are visualized, reducing the risk of vascular injuries and neurological complications [18]. However, the retrojugular approach has limitations, including unfamiliar anatomy, longer operative times, and the risk of phrenic and accessory nerve injuries [5, 18], as well as Horner’s syndrome. The retrojugular approach is preferably used for foraminal lesions with significant foraminal enlargement and ventral extension. It provides direct exposure to the lesion and facilitates early identification of the vertebral artery, as demonstrated in our case.

In the case described by the authors, the foraminal chondroma is removed through a retrojugular approach with a limited anterior cervical foraminotomy to facilitate microsurgical resection of the tumor. Due to an erroneous anatomical localization during surgery, the C6 transverse process is removed resulting in a lesion of the right VA from the use of an ultrasonic bone scalpel. The right VA lesion was repaired using bipolar coagulation according to the “open-close” technique, progressively reducing the defect size to restore vessel wall integrity [6]. Direct visualization of the VA through the retrojugular approach enabled direct control of the accidental arterial bleeding [18].

## Conclusions

Among subaxial foraminal tumors, chondromas should be considered in the differential diagnosis despite their rarity. Complete surgical resection is curative for foraminal chondromas of the subaxial spine, and appropriate approach selection is crucial for satisfactory surgical outcomes. Literature evidence shows that the retrojugular approach offers a valid surgical routed for removing cervical foraminal chondromas, offering direct control of surrounding neurovascular structures and preserving spine stability thus avoiding the need for cervical stabilization.

## Data Availability

No datasets were generated or analysed during the current study.

## References

[CR1] 1. Asazuma T, Toyama Y, Maruiwa H, Fujimura Y, Hirabayashi K (2004) Surgical strategy for cervical dumbbell tumors based on a three-dimensional classification. Spine (Phila Pa 1976) 29:10–14. 10.1097/01.brs.0000103662.13689.76 10.1097/01.BRS.0000103662.13689.76 14699292

[CR2] 2. Baber WW, Numaguchi Y, Kenning JA, Harkin JC (1988) Periosteal chondroma of the cervical spine: one more cause of neural foramen enlargement. Surg Neurol 29:149–152. 10.1016/0090-3019(88)90074-2 10.1016/0090-3019(88)90074-2 3336850

[CR3] 3. Banczerowski P, Veres R, Vajda J (2014) Modified surgical approach to cervical neurinomas with intraforaminal components: minimal invasive facet joint sparing “open-tunnel” technique. J Neurol Surg A Cent Eur Neurosurg 75:16–19. 10.1055/s-0032-1327445 10.1055/s-0032-1327445 23044910

[CR4] 4. Barrey C, Kalamarides M, Polivka M, George B (2005) Cervical dumbbell intra-extradural hemangioblastoma: total removal through the lateral approach: technical case report. Neurosurgery 56:E625; discussion E625 10.1227/01.NEU.0000154134.83900.05 28184666

[CR5] 5. Bobinski L, Henchoz Y, Sandu K, Duff JM (2015) Single stage transforaminal retrojugular tumor resection: The spinal keyhole for dumbbell tumors in the cervical spine. Surg Neurol Int 6:16–19. 10.4103/2152-7806.154453 10.4103/2152-7806.154453 PMC4395986 25883845

[CR6] 6. Choque-Velasquez J, Colasanti R, Jahromi BR, Rafei A, Sharafeddin F, Hernesniemi J (2016) Short-Burst Bipolar Coagulation for Repairing Partially Damaged Brain Arteries Preserving Their Flow: Technical Note. World Neurosurg 93:324–329. 10.1016/j.wneu.2016.06.013 10.1016/j.wneu.2016.06.013 27312393

[CR7] 7. Culver JE, Sweet DE, McCue FC (1975) Chondrosarcoma of the hand arising from a pre existent benign solitary enchondroma: case report and pathological description. Clin Orthop Relat Res. 10.1097/00003086-197511000-00018 10.1097/00003086-197511000-00018 1192657

[CR8] 8. Enneking WF (1986) A system of staging musculoskeletal neoplasms. Clin Orthop Relat Res 9–24 3456859

[CR9] 9. Fahim DK, Johnson KK, Whitehead WE, Curry DJ, Luerssen TG, Jea A (2009) Periosteal chondroma of the pediatric cervical spine. J Neurosurg Pediatr 3:151–156. 10.3171/2008.11.PEDS08231 10.3171/2008.11.PEDS08231 19278317

[CR10] 10. George B, Zerah M, Lot G, Hurth M (1993) Oblique transcorporeal approach to anteriorly located lesions in the cervical spinal canal - Technical note. Acta Neurochir (Wien) 121:187–190. 10.1007/BF01809273 10.1007/BF01809273 8512017

[CR11] 11. Hakuba A, Komiyama M, Tsujimoto T, Ahn MS, Nishimura S, Ohta T, Kitano H (1984) Transuncodiscal approach to dumbbell tumors of the cervical spinal canal. J Neurosurg 61:1100–1106. 10.3171/jns.1984.61.6.1100 10.3171/jns.1984.61.6.1100 6502239

[CR12] 12. Huang Y, Wang Z, Chen Z, Wu H, Jian F (2017) Posterior Hemi-/Laminectomy and Facetectomy Approach for the Treatment of Dumbbell-Shaped Schwannomas in the Subaxial Cervical Spine: A Retrospective Study of 26 Cases. Eur Neurol 78:188–195. 10.1159/000479814 10.1159/000479814 28898892

[CR13] 13. Iwasaki Y, Hida K, Koyanagi I, Yoshimoto T, Abe H (1999) Anterior approach for dumbbell type cervical neurinoma. Neurol Med Chir (Tokyo) 39:835–840. 10.2176/nmc.39.835 10.2176/nmc.39.835 10639809

[CR14] 14. Jeong D, Paeng S (2015) Enchondroma of the cervical spine in young woman: A rare case report. Asian J Neurosurg 10:334–337. 10.4103/1793-5482.162725 10.4103/1793-5482.162725 PMC4558817 26425170

[CR15] 15. Ji W, Cheng Y, Zhu Q, Huang Z, Lin J, Yang D, Ding R, Bao M, Chen J, Jiang H (2021) Posterior unilateral exposure and stability reconstruction with pedicle and lamina screw fixation for the cervical dumbbell tumorectomy: a case report and biomechanical study. European Spine Journal 30:568–575. 10.1007/s00586-020-06668-1 10.1007/s00586-020-06668-1 33219882

[CR16] 16. Jiang L, Lv Y, Liu XG, Ma QJ, Wei F, Dang GT, Liu ZJ (2009) Results of surgical treatment of cervical dumbbell tumors: Surgical approach and development of an anatomic classification system. Spine (Phila Pa 1976) 34:1307–1314. 10.1097/BRS.0b013e3181a27a32 10.1097/BRS.0b013e3181a27a32 19455006

[CR17] 17. Jinnai T, Hoshimaru M, Koyama T (2005) Clinical characteristics of spinal nerve sheath tumors: Analysis of 149 cases. Neurosurgery 56:510–515. 10.1227/01.NEU.0000153752.59565.BB 10.1227/01.neu.0000153752.59565.bb 15730576

[CR18] 18. Lot G, George B (1997) Cervical neuromas with extradural components: Surgical management in a series of 57 patients. Neurosurgery 41:813–822. 10.1097/00006123-199710000-00010 10.1097/00006123-199710000-00010 9316042

[CR19] 19. Lozes G, Fawaz A, Perper H, Devos P, Benoit P, Krivosic I, Jomin M (1987) Chondroma of the cervical spine. Case report. J Neurosurg 66:128–130. 10.3171/jns.1987.66.1.0128 10.3171/jns.1987.66.1.0128 3783244

[CR20] 20. Macnab IAN (1971) Negative Disc Exploration: AN ANALYSIS OF THE CAUSES OF NERVE-ROOT INVOLVEMENT IN SIXTY-EIGHT PATIENTS. JBJS 53 4326746

[CR21] 21. Mangham DC (2004) World Health Organisation classification of tumours: pathology and genetics of tumours of soft tissue and bone. J Bone Joint Surg Br 86-B:466–466. 10.1302/0301-620x.86b3.0860466b

[CR22] 22. McCormick PC (1996) Surgical management of dumbbell tumors of the cervical spine. Neurosurgery 38:294–300. 10.1097/00006123-199602000-00012 10.1097/00006123-199602000-00012 8869056

[CR23] 23. McCormick PC (1996) Surgical management of dumbbell tumors of the cervical spine. Neurosurgery 38:294–300. 10.1097/00006123-199602000-00012 10.1097/00006123-199602000-00012 8869056

[CR24] 24. McLoughlin GS, Sciubba DM, Wolinsky JP (2008) Chondroma/Chondrosarcoma of the Spine. Neurosurg Clin N Am 19:57–63. 10.1016/j.nec.2007.09.007 10.1016/j.nec.2007.09.007 18156048

[CR25] 25. Morard M, De Tribolet N, Janzer RC (1993) Chondromas of the spine: Report of two cases and review of the literature. Br J Neurosurg 7:551–556. 10.3109/02688699308995078 10.3109/02688699308995078 8267893

[CR26] 26. Murad MH, Sultan S, Haffar S, Bazerbachi F (2018) Methodological quality and synthesis of case series and case reports. 23:60–63 10.1136/bmjebm-2017-110853 PMC6234235 29420178

[CR27] 27. Nakamura M, Iwanami A, Tsuji O, Hosogane N, Watanabe K, Tsuji T, Ishii K, Toyama Y, Chiba K, Matsumoto M (2013) Long-term surgical outcomes of cervical dumbbell neurinomas. Journal of Orthopaedic Science 18:8–13. 10.1007/s00776-012-0300-2 10.1007/s00776-012-0300-2 22948961

[CR28] 28. Ozawa H, Kokubun S, Aizawa T, Hoshikawa T, Kawahara C (2007) Spinal dumbbell tumors: An analysis of a series of 118 cases. J Neurosurg Spine 7:587–593. 10.3171/SPI-07/12/587 10.3171/SPI-07/12/587 18074682

[CR29] 29. Palaoglu S, Akkas O, Sav A (1988) Chondroma of the cervical spine. Clin Neurol Neurosurg 90:253–255. 10.1016/0303-8467(88)90032-7 10.1016/0303-8467(88)90032-7 3197353

[CR30] 30. Robles LA, Mundis GM (2021) Chondromas of the Lumbar Spine: A Systematic Review. Global Spine J 11:232–239. 10.1177/2192568220901557 10.1177/2192568220901557 PMC7882828 32875852

[CR31] 31. Sarmiento JM, Medina O, Do AS-MS, Farber S, Chu RM (2019) Resection of Cervical Juxtacortical Chondroma and Circumferential Spinal Stabilization for Kyphotic Deformity. Cureus 11:e4523 10.7759/cureus.4523 PMC6590731 31259132

[CR32] 32. Shurland AT, Flynn JM, Heller GD, Golden JA (1999) Tumor of the cervical spine in an 11-year-old girl [clinical clinical]. Clin Orthop Relat Res 287–290,293–295. 10.1097/00003086-199911000-00035 10.1097/00003086-199911000-00035 10613180

[CR33] 33. Slowik T, Bittner-Manioka M, Grochowski W (1968) Case reports and technical notes. Chondroma of the cervical spine. Case report. J Neurosurg 29:276–279. 10.3171/jns.1968.29.3.0276 10.3171/jns.1968.29.3.0276 5684408

[CR34] 34. Vandenbulcke A, D’Onofrio GF, Capo G, Baassiri W, Barrey CY (2023) Sacrifice of Involved Nerve Root during Surgical Resection of Foraminal and/or Dumbbell Spinal Neurinomas. Brain Sci 13:109. 10.3390/brainsci13010109 10.3390/brainsci13010109 PMC9856955 36672090

[CR35] 35. Verbiest H (1968) A lateral approach to the cervical spine: technique and indications. J Neurosurg 28:191–203. 10.3171/jns.1968.28.3.0191 10.3171/jns.1968.28.3.0191 5643912

[CR36] 36. Willis BK, Heilbrun MP (1986) Enchondroma of the cervical spine. Neurosurgery 19:437–440. 10.1227/00006123-198609000-00017 10.1227/00006123-198609000-00017 3762893

